# Electronegative Strategic Positions in Covalent Organic Frameworks: Unlocking High‐Efficiency Gold Recovery

**DOI:** 10.1002/anie.202502199

**Published:** 2025-03-18

**Authors:** Zhongping Li, Wanyi Zhao, Changqing Li, Yawei Yin, Dongxue Wei, Yucheng Jin, Yongfeng Zhi, Jikuan Qiu, Yuwei Zhang, Jong‐Beom Baek

**Affiliations:** ^1^ School of Materials Science and Engineering Jilin University Changchun 130012 P.R. China; ^2^ Department of Energy and Chemical Engineering/Center for Dimension‐Controllable Organic Frameworks Ulsan National Institute of Science and Technology (UNIST) Ulsan 44919 Republic of Korea; ^3^ Department Laboratory of Preparation and Applications of Environmental Friendly Materials (Jilin Normal University) Ministry of Education Jilin Normal University Changchun 130103 P.R. China; ^4^ College of Chemical Engineering and Technology Hainan University Haikou 570228 P.R. China

**Keywords:** Au capture, Covalent organic frameworks, Electronegative skeleton, Electrostatic interaction, Hexaazatriphenylene

## Abstract

Gold (Au) concentrations accumulated from electronic waste (e‐waste) and industrial leachates far surpass those found in natural ores, a highly valuable resource if efficient recovery methods can be developed. Despite advancements in covalent organic frameworks (COFs), achieving adsorbents with high selectivity, large capacity, and rapid adsorption kinetics remain challenging because of limitations in partial pore wall sites. Here, we present hexaazatriphenylene‐based COFs (HATP‐COFs) with an electronegative skeleton, specifically designed for selective Au recovery. The hexaazatriphenylene centers, imine linkages, and pyridine linkers within the COFs introduce electron‐rich sites that extend across strategic positions—vertex, linkages, and linkers—thereby enhancing the overall structural integrity. These features facilitate efficient Au capture through electrostatic interactions, achieving an exceptional adsorption capacity exceeding 2366 mg g^−1^ with rapid kinetics, making HATP‐COFs one of the most efficient pure COFs reported to date. Moreover, these HATP‐COFs demonstrate remarkable selectivity, stability, and scalability. Theoretical calculations reveal that the electronegative skeleton introduces critical binding sites, promoting strong electrostatic interactions with Au^3+^ ions and improving adsorption kinetics. This work highlights the potential of charge‐interface engineering in COFs as a transformative strategy for developing next‐generation materials.

## Graphical abstract

We present hexaazatriphenylene‐based COFs with an electronegative skeleton designed for selective gold recovery. The hexaazatriphenylene centers, imine linkages, and pyridine linkers generate electron‐rich sites at strategic positions—vertex, linkages, and linkers—thereby enhancing the structural integrity of the framework. These features enable efficient gold capture, providing high‐adsorption capacity and fast kinetics through electrostatic interactions, making the COFs highly effective for selective gold recovery.

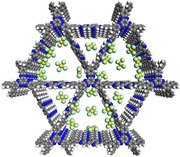

## Introduction

Gold (Au) is a highly valued precious metal because of its exceptional chemical and physical properties.^[^ ^1^, ^2^ ^]^ Widely used in electronics, catalysis, and jewelry, there has been increasing demand for gold, highlighting the need for efficient production and recovery methods.^[^ ^3^, ^4^, ^5^ ^]^ Leachates and electronic waste (e‐waste) contain approximately 2000 ppm of Au, a concentration significantly higher than that found in natural ores, which typically contain around 30 ppm.^[^ ^6^ ^]^ However, both natural ores and e‐waste often contain competing metals such as copper, cadmium, chromium, aluminum, and cobalt, complicating the extraction process, making separation challenging and energy‐intensive.^[^ ^7^, ^8^, ^9^ ^]^ Accordingly, developing selective, reusable Au adsorbents with high capture efficiency and capacity is crucial.^[^ ^10^, ^11^, ^12^ ^]^

Covalent organic frameworks (COFs), an emerging class of crystalline porous organic materials,^[^ ^13^, ^14^, ^15^, ^16^, ^17^, ^18^, ^19^, ^20^ ^]^ offer significant potential for efficient Au recovery due to their unique structural features. COFs are composed of modular building blocks interconnected by covalent bonds, resulting in a well‐defined and highly porous framework.^[^ ^21^, ^22^, ^23^, ^24^, ^25^, ^26^ ^]^ The inherent properties of COFs, such as their high porosity, excellent chemical stability, and tunable functionalities, make them particularly promising for a wide range of applications, including molecular adsorption, catalysis, chemical sensing, and energy storage.^[^ ^26^, ^27^, ^28^, ^29^, ^30^, ^31^, ^32^, ^33^, ^34^, ^35^, ^36^, ^37^, ^38^, ^39^, ^40^ ^]^

Among COF structures, 2D‐COFs are notable for their structural versatility and suitability for adsorption applications. Their tunable structure allows for high porosity, which enhances adsorption capacity, while their ordered 1D pores provide abundant interaction sites for metal cations.^[^ ^28^, ^33^, ^41^, ^42^, ^43^ ^]^ COFs tailored for Au capture can be functionalized with groups, including thiol, phenolic, and amide, which enhance pore‐wall interactions and facilitate Au adsorption. However, despite significant advancements, the performance of COF adsorbents is often constrained by limited partial sites on the pore walls,^[^ ^44^, ^45^, ^46^, ^47^, ^48^, ^49^ ^]^ which hinder selectivity, capacity, and adsorption kinetics. Addressing these limitations is essential to achieving COFs with high‐capacity, rapid gold capture capabilities, and enabling their broader adoption in industrial applications.

**Figure 1:**
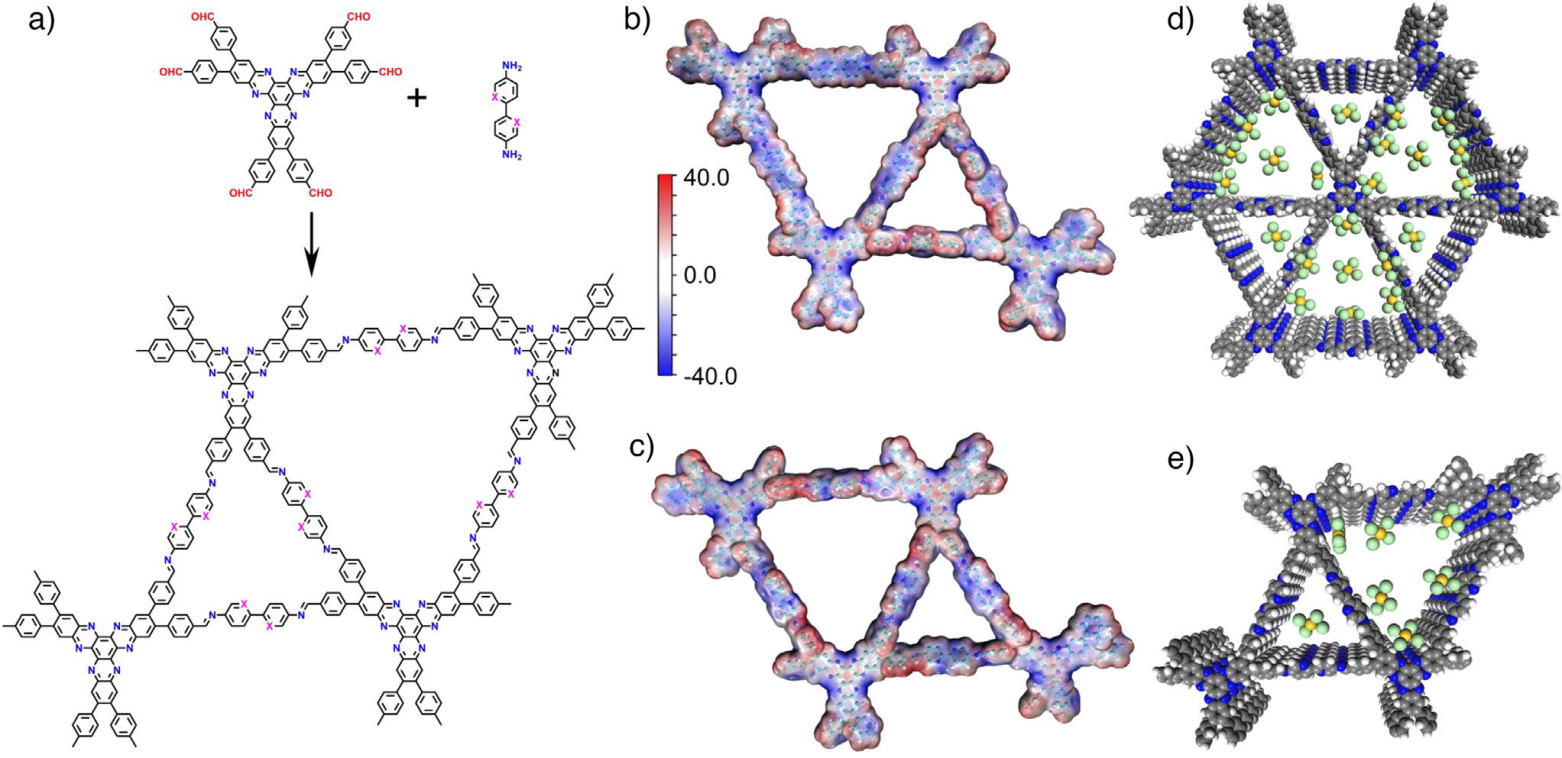
a) Design and synthesis of HATP‐COF‐1 (X = H) and HATP‐COF‐2 (X = N); corresponding electrostatic potential mapping of the surfaces of b) HATP‐COF‐1 and c) HATP‐COF‐2, respectively; d), e) gold‐loaded pore channels of HATP‐COF‐2 shown in five layers.

To address these challenges, we introduce hexaazatriphenylene‐based COFs (HATP‐COFs) with electronegative 1D channels. The incorporation of hexaazatriphenylene centers, imine linkages, and pyridine linkers within the COF skeleton results in electron‐rich sites that extend from the vertex to the walls of the framework (Figure 1a–c). These structural features are critical for facilitating Au capture through electrostatic interactions within the channels, thereby significantly increasing the number of accessible binding sites for Au ions (Figure 1d,e). As a result, these COFs exhibit remarkable adsorption kinetics, exceptional selectivity, and a high capacity for Au recovery. This study emphasizes the potential of incorporating electronegative components at strategic positions—vertex, linkages, and linkers—to develop stable and efficient COFs. These findings open new avenues for transformative applications in selective adsorption, particularly in Au recovery, as well as other areas requiring advanced material design.

## Results and Discussion

The hexaazatriphenylene‐based COFs (HATP‐COF‐1 and HATP‐COF‐2) were respectively synthesized using aldehyde‐modified hexaazatriphenylene as the vertex and either [1,1′‐biphenyl]‐4,4′‐diamine or 5,5′‐diamino‐2,2′‐bipyridine as the linker, as illustrated in Figure 1a. The electrostatic potential (ESP) mapping of the surface, as shown in Figure 1b,c, revealed the distribution of electronegative walls within the HATP‐COFs skeleton. Notably, HATP‐COF‐2 displays a higher density of electronegative sites compared to HATP‐COF‐1, highlighting its superior structural features. Fourier‐transform infrared (FT‐IR) spectroscopy confirmed the presence of carbon nitrogen double bonds associated with the vertex, linkages, and linkers, observed between 1603 and 1698 cm^−1^ for the HATP‐COFs (Figure S1). Additionally, solid‐state ^13^C magic angle spinning (MAS) nuclear magnetic resonance (NMR) spectroscopy provided further structural insights, showing characteristic carbon peaks at 108.3 and 117.5 ppm for the HATP‐COFs (Figure S2). Thermogravimetric analysis (TGA) demonstrated the outstanding thermal stability of these HATP‐COFs under a nitrogen atmosphere (Figure S3). The morphology of the as‐synthesized COFs was investigated using field‐emission scanning electron microscopy (FE‐SEM), revealing their rod‐shaped structural features (Figure S4). Elemental mapping via energy‐dispersive X‐ray spectroscopy (EDS) showed a uniform distribution of carbon and nitrogen elements across the COFs, further corroborating their homogeneous composition and morphology (Figure S5). The X‐ray photoelectron spectroscopy (XPS) spectra provide information on the C 1s and N 1s signals (Figure S6). The elemental composition of HATP‐COFs, including carbon, nitrogen, and hydrogen, was determined experimentally and found to be in close agreement with the theoretical values (Table S1).

**Figure 2:**
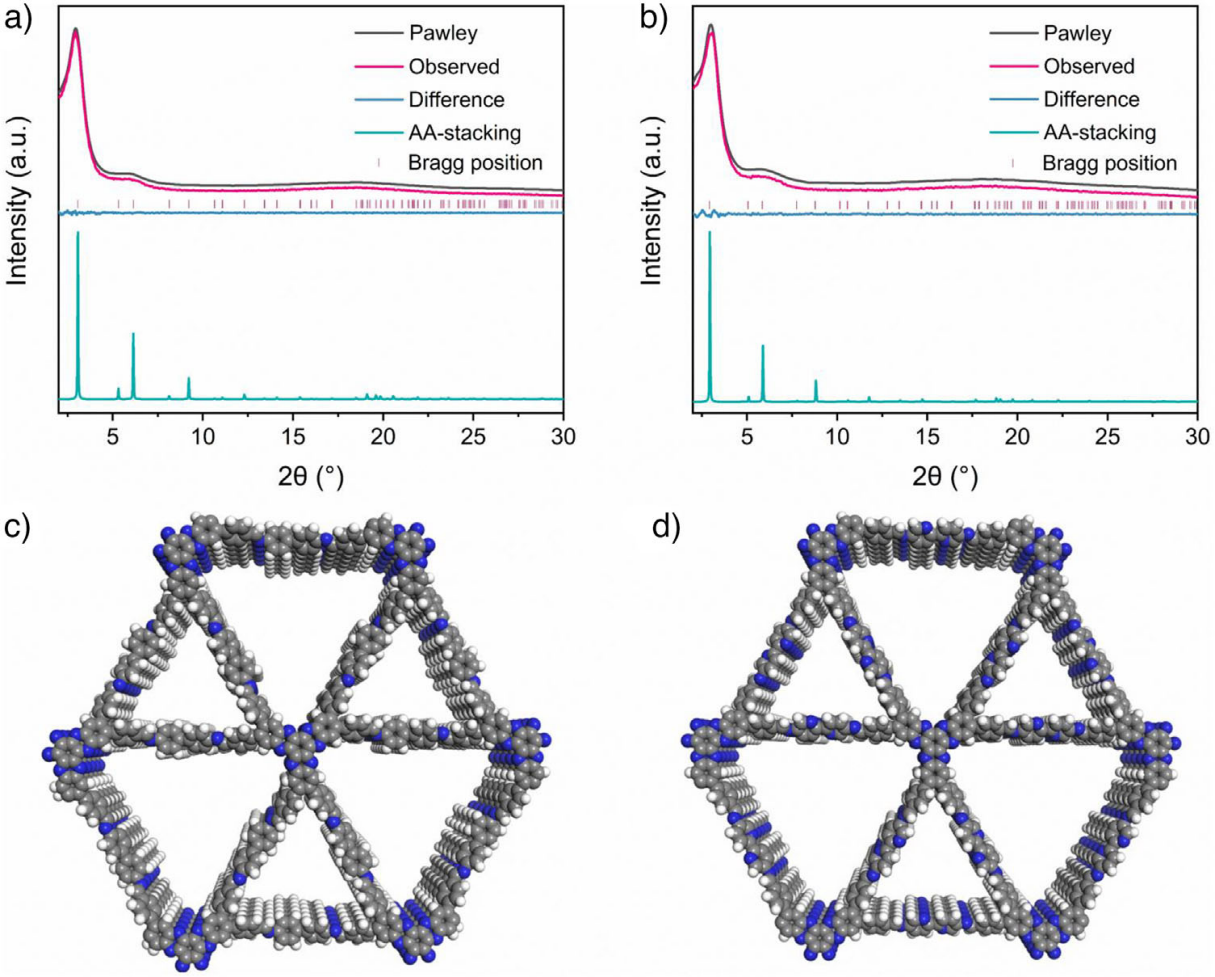
PXRD patterns of a) HATP‐COF‐1 and b) HATP‐COF‐2. Unit crystal cells of AA‐stacking model c) HATP‐COF‐1 and d) HATP‐COF‐2.

The crystal structures of the as‐synthesized HATP‐COFs were analyzed using powder X‐ray diffraction (PXRD). The diffraction patterns confirmed good crystallinity, with prominent crystallization peaks observed in Figure 2a,b. Pawley refinements further revealed that the theoretical structures were consistent with the experimental results and aligned with an AA‐stacking model (Figure 2c,d), while showing no match with the AB‐stacking model (Figures S7 and S8). For HATP‐COF‐1, the refined crystal structure adopted the *P*3 space group, with lattice parameters of *a* = 33.22473 Å, *b* = 33.22473 Å, *c* = 4.70489 Å, and *α* = *β* = *γ* = 90° (Table S2). The refinement achieved good agreement factors, with *R*_wp_ and *R*_p_ values of 1.76% and 1.26%, respectively. Similarly, HATP‐COF‐2 also crystallized in the *P*3 space group, exhibiting identical lattice parameters and refinement factors (*a* = 34.71496 Å, *b* = 34.71496 Å, *c* = 4.71199 Å, and *α* = *β* = *γ* = 90°, *R*_wp_ = 1.80%, *R*_p_ = 1.44%, Table S3). The observed crystallinity and the well‐defined unit cells of the HATP‐COFs further support their structural integrity and potential for predictable behavior in subsequent applications.

To investigate the intrinsic porosity of the HATP‐COFs, nitrogen adsorption–desorption isotherms were measured at 77 K (Figures S9a and S10a). The analyses revealed high Brunauer–Emmett–Teller (BET) surface areas of 1593 and 1667 m^2^ g^−1^ for HATP‐COF‐1 and HATP‐COF‐2, respectively, underscoring their substantial porosity. Furthermore, pore size distribution analyses conducted using the density functional theory (DFT) model demonstrated that the pore sizes of HATP‐COF‐1 (Figure S9b) and HATP‐COF‐2 (Figure S10b) were predominantly centered at 1.1 and 2.0 nm, respectively. These well‐defined pore architectures suggest that the HATP‐COFs possess a uniform and tunable porosity, which is critical for applications.

The chemical and thermal stabilities of the HATP‐COFs were thoroughly assessed by exposing the material to harsh chemical conditions, including dimethylformamide, 1 M aq. HCl, and 1 M aq. NaOH, over a 24‐h period. The crystalline structures of HATP‐COFs remained unchanged under all tested conditions, as confirmed by the PXRD patterns (Figure S11), which showed no significant deviations from the original profile. Furthermore, FT‐IR spectroscopy (Figure S12) corroborated the stability of key functional groups, with no evidence of degradation. These good chemical and thermal stabilities underscore the robustness of HATP‐COFs, highlighting their suitability for applications in harsh environments. This resilience positions the HATP‐COFs as ideal candidates for a wide range of industrial and environmental applications where durable materials are essential.

**Figure 3:**
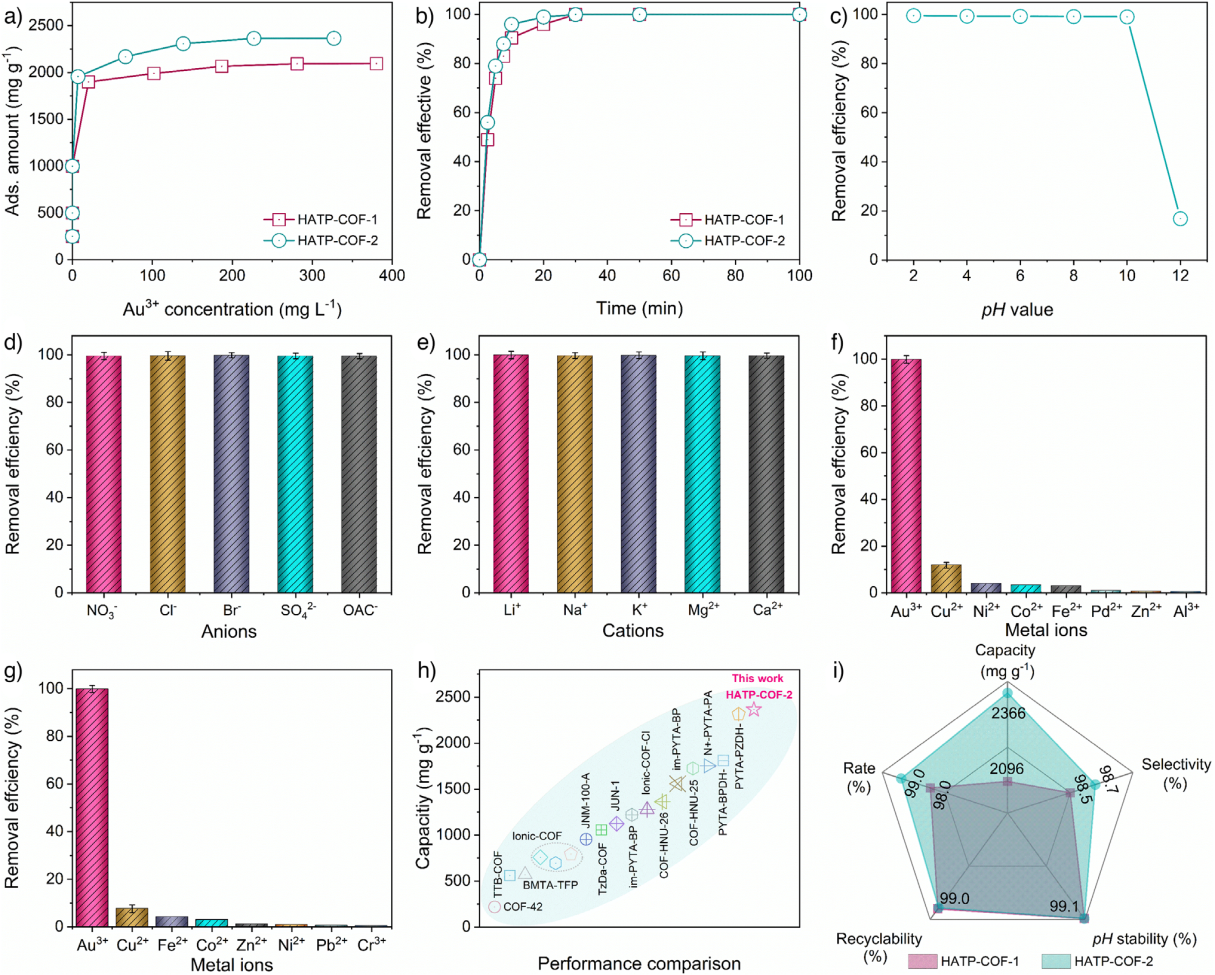
a) Au^3+^ ions adsorption isotherms and b) removal efficiency of Au^3+^ ions for by HATP‐COFs. c) Removal efficiency of Au^3+^ ions for HATP‐COF‐2 under different pH conditions and d) removal efficiency of Au^3+^ ions for HATP‐COF‐2 from solutions containing mixed anions. e) Removal efficiency for alkali and alkaline metal ions, f) removal efficiency for metal and transition metal ions, and g) capture efficiency of metal ions using HATP‐COF‐2 from CPUs. h) Capacity of HATP‐COF‐2 compared to reported COFs. i) Radar plot showing a comparison of different capture parameters for HATP‐COFs under ideal conditions. The error bars represent the standard deviation of three independent experiments performed in triplicate.

The HATP‐COFs, with electronegative skeletons extending from the vertex to the wall, demonstrate significant potential as effective platforms for Au^3+^ ions adsorption. The Au^3+^ ions adsorption behavior of these COFs was evaluated at room temperature, as shown in Figure 3. The equilibrium curves illustrate the adsorption capacities of HATP‐COFs at initial Au^3+^ ions concentrations ranging from 0 to 800 mg L^−1^. HATP‐COF‐1 and HATP‐COF‐2 achieved equilibrium adsorption capacities of 2096 and 2365 mg g^−1^, respectively (Figure 3a,i), marking some of the excellent performance values reported for COFs to date (Figure 3h).^[^ ^22^, ^45^, ^46^, ^47^, ^48^, ^49^, ^50^, ^51^, ^52^, ^53^, ^54^, ^55^, ^56^, ^57^, ^58^, ^59^ ^]^ To further elucidate the adsorption mechanism, the equilibrium data were fitted to both the Langmuir and Freundlich adsorption isotherm models (Figures S13 and S14). The Langmuir model provided a higher correlation coefficient (*R* ^2^ > 0.999) compared to the Freundlich model, suggesting that the adsorption process follows the Langmuir isotherm and is best described by a monolayer adsorption mechanism.

Kinetic studies were conducted with an initial Au^3+^ ions concentration of 200 mg L^−1^ in a 100 mL solution. The data were analyzed using both the pseudo‐first‐order and pseudo‐second‐order kinetic models (Figures S15 and S16). The pseudo‐second‐order model provided a better fit, with a high correlation coefficient (*R* ^2^ > 0.999), suggesting that the Au^3+^ ions adsorption onto the HATP‐COFs is primarily governed by the availability of electronegative adsorption sites, which are distributed from the vertex to the wall.

As shown in Figure 3b, HATP‐COF‐2 exhibited a rapid adsorption rate, achieving 99% Au^3+^ ions removal within 10 min, outperforming HATP‐COF‐1, which reached 90% removal. After 20 min, both HATP‐COFs had almost completely removed all Au^3+^ ions from the solution. This ultrafast adsorption capability can be attributed to the inherent electronegative adsorption sites integrated into the ordered COF structure, rather than modifications to the channel structures.

Stability and selectivity are crucial factors for adsorption applications. Considering that practical gold recovery often involves acidic conditions due to the necessity of digesting e‐waste with acid solutions, we evaluated the gold capture efficiency of HATP‐COFs across a pH range of 2–12 (Figures 3c and S17). Notably, the HATP‐COFs demonstrated exceptional performance, capturing over 99% of Au^3+^ ions within a pH range of 2–10. However, their uptake efficiency significantly decreased to 17% in alkaline conditions. These findings suggest that HATP‐COFs are highly effective for Au^3+^ ions adsorption over a broad pH range, making them suitable for practical applications in acidic, neutral, and weakly alkaline environments.

To evaluate the selectivity of HATP‐COFs in the presence of competing anions, adsorption experiments were conducted using solutions containing various anions, including NO_3_^−^, SO_4_^2−^, OAc^−^, and Cl^−^ (Figures 3d and S18). The HATP‐COFs demonstrated consistently high adsorption efficiencies, underscoring their robustness in practical applications. Similarly, their adsorption capacities for alkali and alkaline earth metals remained largely unaffected (Figures 3e and S19).

The selective extraction of Au^3+^ ions was further assessed in complex leaching solutions containing competing metal ions such as Cu^2+^, Ni^2+^, Co^2+^, Fe^2+^, Pd^2+^, Zn^2+^, and Al^3+^. The results indicated there was no significant reduction in Au adsorption capacity (Figures 3f and S20). For practical validation, HATP‐COFs were applied to a leachate derived from discarded central processing units (CPUs), and achieved an Au^3+^ ions capture efficiency exceeding 98% (Figures 3g and S21). In contrast, the adsorption of other metal ions was minimal, remaining below 10%. These findings collectively highlight the excellent selectivity of HATP‐COFs for Au adsorption, even in the presence of competing anions and metal ions, affirming their potential as highly effective adsorbents for practical gold recovery in complex environments.

The reusability of Au adsorbents is a critical parameter for evaluating their practical applicability. To investigate the cyclic performance of HATP‐COFs for Au^3+^ ions adsorption, we conducted multiple adsorption–desorption cycles. After each adsorption cycle, the Au^3+^‐loaded HATP‐COFs were regenerated using thiourea as an eluent for 60 min over several cycles. This process leverages the strong binding affinity between Au and thiourea, facilitating efficient desorption. The regenerated HATP‐COFs were subsequently reused for Au^3+^ ions adsorption. Even after five cycles, the HATP‐COFs retained an adsorption efficiency exceeding 98%, demonstrating outstanding reusability and consistent performance (Figure S22).

To further assess the structural and chemical stability of the HATP‐COFs after repeated cycling, PXRD analysis was performed. The results indicated that the regenerated HATP‐COFs preserved their crystallinity, with marginal changes compared to the original material (Figure S23). FT‐IR spectroscopy revealed no significant alterations in the functional groups of the HATP‐COFs, confirming their chemical stability throughout the cycling process (Figure S24). Additionally, FE‐SEM images show that the COFs maintained a consistent rod‐like morphology with the previously synthesized COFs (Figures S4 and S25).

Overall, these results establish HATP‐COFs as an outstanding material for Au^3+^ ions adsorption, achieving a remarkable balance of capacity, efficiency, durability, selectivity, and reusability. This combination of properties positions HATP‐COFs as a leading candidate for sustainable and cost‐effective gold recovery in industrial and environmental applications.

To elucidate the mechanism underlying the ultrahigh Au recovery observed in hexaazatriphenylene‐based COFs, we conducted comprehensive experimental and theoretical analyses to investigate their binding energies, structural interactions, and electronic properties. XPS analysis of Au‐loaded HATP‐COFs (Au@HATP‐COFs) revealed the binding energies after Au incorporation (Figures S26 and S27). N 1s speactra of Au@HATP‐COFs observed the emergence of Au─N (401.2 eV) and C═N peaks (398.3 eV), substantiating our stategy of incorporating hexaazatriphenylene centers, imine linkages, and pyridine linkers within the COF skeleton for improved interactions with target metal ions. For the Au 4f orbital, XPS spectra exhibited peaks at 84.7 and 88.6 eV, corresponding to metallic Au(0). Additional pair peaks appeared at around 86.8 and 91.3 eV were attributed to the presence of Au(III), reflecting the multifaceted interactions occurring during the gold capture process. PXRD analysis further confirmed successful gold capture, as evidenced by the appearance of diffraction peaks at 38.1°, 44.3°, 64.5°, 77.4°, and 81.6°, corresponding to the (111), (200), (220), (311), and (222) crystal facets of Au(0), respectively (Figure S28). Moreover, EDX mapping confirmed the distribution of Au throughout the HATP‐COFs (Figures S29 and S30), highlighting the effectiveness of the COF design for metal ions adsorption.

**Figure 4:**
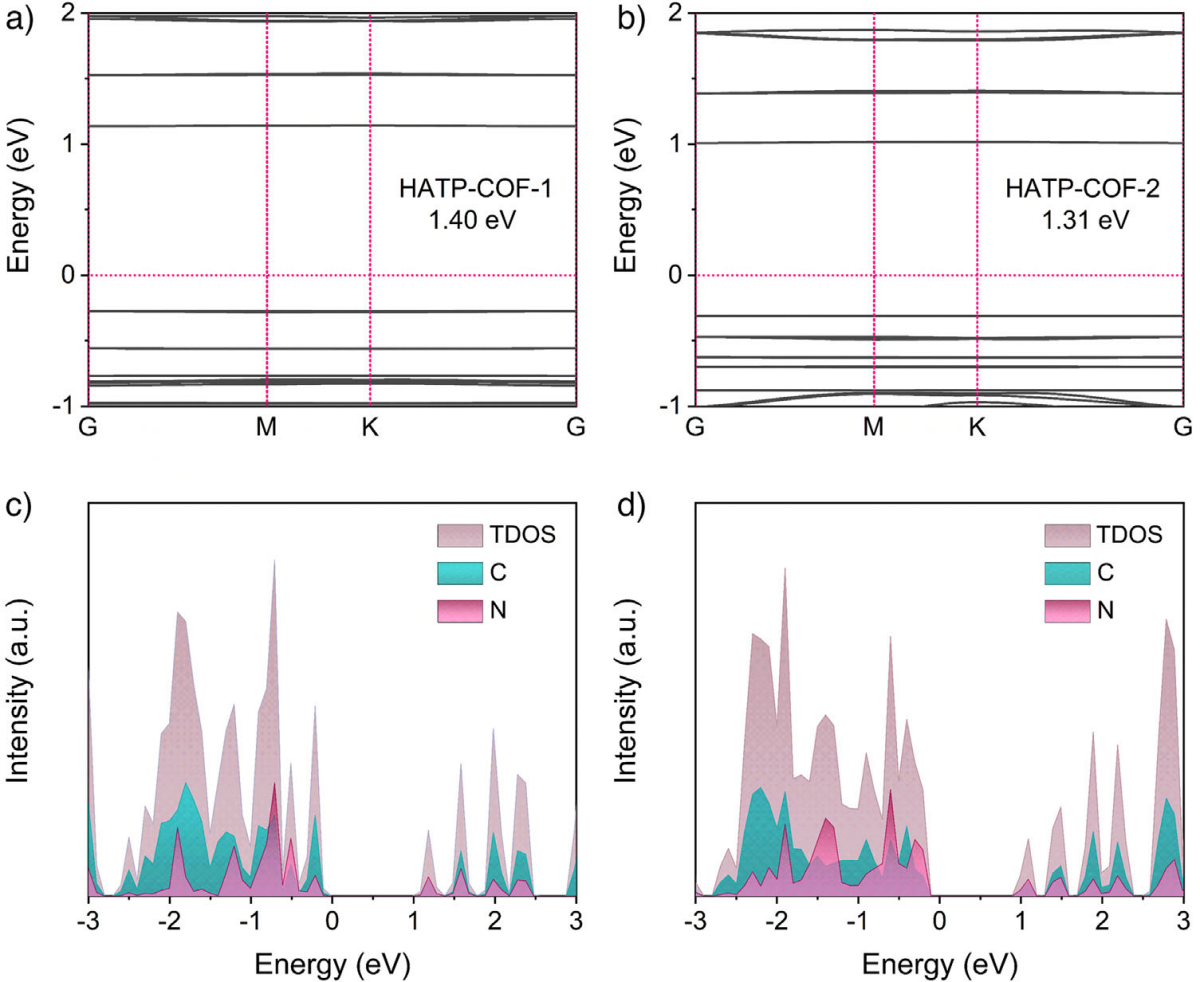
Calculated band structures of a) HATP‐COF‐1 and b) HATP‐COF‐2. Density of states of c) HATP‐COF‐1 and d) HATP‐COF‐2.

To complement the experimental findings, theoretical calculations were conducted to investigate the electron transfer and binding energies of HATP‐COFs. Density of states (DOS) analyses revealed that HATP‐COF‐2 exhibited a lower band gap of 1.31 eV compared to 1.40 eV for HATP‐COF‐1 (Figure 4a,b), indicating stronger interactions between the COF skeleton and Au^3+^ ions, which facilitates efficient electron transfer. The projected density of states analysis demonstrated that the primary contributions to the electronic structure originate from carbon and nitrogen atoms (Figure 4c,d). Additionally, the nitrogen atoms in HATP‐COF‐2 showed a broader distribution near the Fermi level, which likely enhances interactions with Au^3+^ ions. These findings correspond to the experimentally observed high Au recovery performance, offering deeper insights into the structural and electronic factors that contribute to the exceptional capabilities of HATP‐COFs.

**Figure 5:**
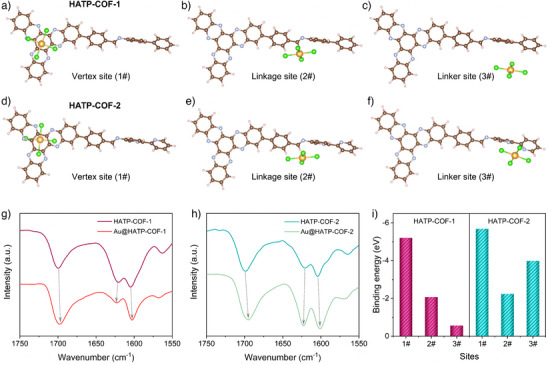
Au^3+^ ions binding energy at vertex sites of a) HATP‐COF‐1 and d) HATP‐COF‐2, linkage sites of b) HATP‐COF‐1 and e) HATP‐COF‐2, and linker sites of c) HATP‐COF‐1 and f) HATP‐COF‐2. FT‐IR spectra of g) HATP‐COF‐1 and Au@HATP‐COF‐1, and h) HATP‐COF‐2 and Au@HATP‐COF‐2. i) Comparison of Au^3+^ ions binding energies at different sites of the HATP‐COFs with Au^3+^ ions.

The binding energy analysis highlighted the significance of the strategically positioned electronegative sites at the vertex, linkages, and linkers within the HATP‐COFs. For HATP‐COF‐1, binding energies were approximately −5.21 eV at the hexaazatriphenylene centers (Figure 5a,i) and −2.07 eV at the linkages (Figure 5b,i). In contrast, HATP‐COF‐2 exhibited strong binding energies of −5.69 and −2.24 eV (Figure 5d,e,i), respectively. Notably, the electronegative linker in HATP‐COF‐2 demonstrated a significantly higher binding energy of −3.99 eV (Figure 5f,i), approximately seven times greater than that of the neutral linker in HATP‐COF‐1 (Figure 5c,i). These findings were further supported by FT‐IR results, which revealed notable shifts in the C═N bond, confirming strong interactions between the electronegative sites in the COFs and Au^3+^ ions (Figure 5g,h). The enhanced binding energies, coupled with an increased number of effective binding sites, account for the superior Au capture capacity of HATP‐COF‐2. The additional electronegative sites within the linker regions further strengthen the affinity for Au^3+^ ions, facilitating more effective interactions. Therefore, functionalizing the vertex, linkages, and linkers with electronegative groups emerges as a highly effective strategy for optimizing COF performance in metal recovery applications. This approach not only enhances selectivity and efficiency but also represents a significant step toward the development of scalable and sustainable technologies for metal ion capture.

## Conclusion

In conclusion, this study presents hexaazatriphenylene‐based COFs with an electronegative skeleton designed for selective Au recovery. The frameworks’ hexaazatriphenylene centers, imine linkages, and pyridine linkers create electron‐rich sites at strategic positions—vertex, linkages, and linkers—enhancing the overall structural integrity. These features enable efficient Au capture through electrostatic interactions, resulting in exceptional adsorption capacity and rapid kinetics, making HATP‐COFs among the most efficient COFs for Au recovery. Additionally, the tested HATP‐COFs demonstrated high selectivity, stability, and scalability, positioning them for large‐scale applications. Theoretical calculations further confirmed that the electronegative skeleton provides critical binding sites that promote strong interactions with Au^3+^ ions and improve adsorption kinetics. This work highlights the potential of charge‐interface engineering in COFs as a strategy for designing next‐generation materials, contributing to sustainable metal recovery and advancing COF applications in environmental and industrial contexts.

## Author Contributions

Z.L, W.Z, Y.J, and C.L performed the primary experiments and data collection. Y.J. was responsible for the theoretical calculations. Supporting experiments were conducted by Y.Y., D.W., J.Q., and Y.Z. Z.L., C.L., Y.Z., Y.J., and J.‐B.B. wrote the manuscript, and all authors discussed the results and provided comments on the manuscript.

## Conflict of Interests

The authors declare no conflict of interest.

## Supporting information



Supporting Information

## Data Availability

The data that support the findings of this study are available from the corresponding author upon reasonable request.
