# Supplementary material for: Electronegative Strategic Positions in Covalent Organic Frameworks: Unlocking High‐Efficiency Gold Recovery
Source: Angew Chem Int Ed Engl. 2025 Mar 18;64(19):e202502199. doi: 10.1002/anie.202502199 (PMC12051782; doi:10.1002/anie.202502199)
Supplement: Supplementary file 1 — Supporting Information [file ANIE-64-e202502199-s001.docx]

**Electronegative Strategic Positions in Covalent Organic Frameworks: Unlocking High-Efficiency Gold Recovery**

**Materials**

The mesitylene acetic acid, DMF, DMAc, tetrahydrofuran, acetone, chloroauric acid and so on were obtained from Tokyo Chemical Industry Co., Ltd., Sigma Aldrich, and Samchun Chemical Co., Ltd. 4,4',4'',4''',4'''',4'''''-(Diquinoxalino[2,3-a:2',3'-c]phenazine-2,3,8,9,14,15-hexayl)hexabenzaldehyde and 5,5'-diamino-2,2'-bipyridine were obtained from Jilin Zhongke Yanshen Technology Co., Ltd.

**Characterization**

Fourier transform infrared (FT‑IR) spectra were recorded on a Perkin‑Elmer Spectrum 100 spectrometer by using KBr pellets. UV-visible absorption spectra were recorded on a Cary 5000 spectrometer equipped with a D2 lamp. Solid state cross polarization magic-angle spinning nuclear magnetic resonance (ss CP-MAS NMR) spectra were recorded on an Agilent VNMRS 600 spectrometer at a spinning frequency of 10 kHz. Thermogravimetric analysis (TGA) measurements were conducted on an STA 8000 (Perkin-Elmer). The sample was heated from 50 to 900 °C at a heating rate of 10 °C min^−1^ under a nitrogen atmosphere. Element contents were recorded by PerkinElmer NexION 350X ICP-MS equipment. Powder X‑ray diffraction patterns were collected using a Rigaku D/Max 2500 rotating anode X‑ray powder diffractometer using Cu Kα radiation (λ = 1.5406 Å) operated at 1600 W (40 kV, 40 mA) power and equipped with a position sensitive detector with a 10.0 mm divergence height slit. Nitrogen sorption isotherms were recorded on a BELSORP‑max at 77 K. Prior to the measurements of the sorption isotherms, the samples were degassed for 24 h at 120 °C under high vacuum. The calculations of the pore size distribution were performed using the nonlocal density functional theory (NLDFT) adsorption model with cylindrical pores. High resolution transmission electron microscopy (HR-TEM) was performed with a JEOL JEM‑2100F equipped with a field emission gun operated at 200 kV. Field emission scanning electron microscopy (SEM) images were obtained using a Hitachi SU8220 Cold FE-SEM scanning electron microscope.

**Synthesis of COFs**

**Synthesis of HATP-COF-1**: A Pyrex tube (16 mL) was charged with [1,1'-biphenyl]-4,4'-diamine (0.30 mmol) and 4,4',4'',4''',4'''',4'''''-(diquinoxalino[2,3-a:2',3'-c]phenazine-2,3,8,9,14,15-hexayl)hexabenzaldehyde (0.10 mmol), 1.60 mL DMAc/Mesitylene (v/v, 3:1), and 0.2 mL of 6 M aqueous acetic acid. The tubes were sonicated for two mins, flash frozen in liquid nitrogen bath and degassed by three freeze-pump-thaw cycles. The tubes were sealed off and heated at 120 °C for three days. The powder collected was washed with DMF, tetrahydrofuran, acetone and methanol several times, Soxhleted by acetone overnight, and then dried at 100 °C under vacuum for 12 h to obtain HATP-COF-1 (Yield: 89%).

**Synthesis of HATP-COF-2**: A Pyrex tube (16 mL) was charged with 5,5'-diamino-2,2'-bipyridine (0.30 mmol) and 4,4',4'',4''',4'''',4'''''-(diquinoxalino[2,3-a:2',3'-c]phenazine-2,3,8,9,14,15-hexayl)hexabenzaldehyde (0.10 mmol), 1.60 mL DMAc/Mesitylene (v/v, 3:1), and 0.2 mL of 6 M aqueous acetic acid. The tubes were sonicated for two mins, flash frozen in liquid nitrogen bath and degassed by three freeze-pump-thaw cycles. The tubes were sealed off and heated at 120 °C for three days. The powder collected was washed with DMF, tetrahydrofuran, acetone and methanol several times, Soxhleted by acetone overnight, and then dried at 100 °C under vacuum for 12 h to obtain HATP-COF-2 (Yield: 84%).

**Preparation and adsorption procedure for Au^3+^ sorption**

Aqueous solutions with varying concentrations of gold ions (Au^3+^) were prepared. Subsequently, 2 mg of covalent organic frameworks (COFs) was added to 10 mL of Au^3+^ solution at room temperature and stirred for 12 hours. The mixtures were filtered using a 0.2 μm membrane filter. The gold concentration in the filtrates was measured via inductively coupled plasma-optical emission spectroscopy (ICP-OES).

The equilibrium adsorption capacity, q_e_ (mg g^–1^), was calculated using the equation:

q_e_=(C_0_-C_e_)×V/m

where C_0_ and Ce​ (mg L^–1^) are the initial and equilibrium concentrations of Au^3+^, V (L) is the solution volume, and m (g) is the mass of COFs

**Kinetic study of Au³⁺ sorption**

An aqueous solution containing 200 mg L^–1^ of Au^3+^ was prepared. A total of 10 mg of COFs was added to 100 mL of the Au^3+^ solution and stirred for 100 min. At predetermined intervals, aliquots of the mixture were withdrawn, filtered using a 0.2 μm membrane, and analyzed for gold concentration via ICP-OES.

The capture efficiency (R_e_, %) was determined using:

R_e_=(C_0_-C_e_)/C_0_

where C_0_​ and C_e_ ​are the initial and equilibrium Au³⁺ concentrations, respectively.

**Anion/ions selectivity tests**

**Test 1**: A solution containing NaNO_3_, Na_2_SO_4_, NaBr, NaOAc, and NaCl (each 200 mg L^–1^) along with 200 ppm of Au^3+^ was prepared. A 10 mg sample of COFs was added to 10 mL of the mixture, stirred at room temperature for 12 hours, filtered using a 0.2 μm membrane, and analyzed via ICP-OES. **Test 2**: A mixture solution containing Li^+^, Na^+^, K^+^, Ca^2+^, Mg^2+^, and Au^3+^ (200 mg L^–1^ each) was prepared. The procedure for adsorption and analysis was identical to Test 1. **Test 3**: A mixture solution containing metal ions (Fe^2+^, Co^2+^, Ni^2+^, and so on) and Au^3+^ (200 mg L^–1^ each) was prepared. The procedure for adsorption and analysis followed the same steps as Test 1.

**Preparation of CPU leaching solution**

Central processing units (CPUs) were soaked in 10 mL of aqua regia for 24 hours to produce an initial solution. The solution's pH was adjusted to 2 and filtered through a 0.2 μm membrane, resulting in the final leaching solution with a pH of 2.

**Recycling Experiments**

Aqueous solutions containing 200 mg L^–1^ of Au^3+^ were prepared. A 30 mg sample of COFs was added to 100 mL of Au^3+^ solution and stirred for 12 hours. The COF-Au composite was filtered, washed with water three times, and subsequently treated with a regenerating solution (0.01 mol L^–1^ thiourea and 0.05 mol L^–1^ HCl) for 12 hours. The regenerated COFs were filtered and washed with water three times before reuse.

All adsorption experiments were repeated three times.

**Stability measurements**

The HATP-COF samples were dispersed in water, dimethylformamide, and aqueous NaOH and HCl solutions at room temperature for 24 hours. The samples were then collected via simple filtration and washed sequentially with THF, water, and acetone. Under acidic conditions, the samples were washed three times with ammonia water. Under basic conditions, they were washed three times with a small amount of acetic acid followed by a large volume of water. Finally, the HATP-COF samples were dried under vacuum at 70 °C for 12 hours.

**Computation details**

A comprehensive exploration of the binding affinities at various sites, specifically nitrogen within tetrazine and sulfur within tetrafulvalene, with [AuCl_4_]^–1^ has been undertaken through quantum chemical computations. The quasi-Newton algorithm was applied for energy ensemble minimization, using a convergence tolerance of 0.005 kcal (mol⋅Å)^–1^ for force and 5 × 10^–5^ Å for displacement. A spatial extension of approximately 70 Å in the *z*-axis was introduced to mitigate the impact of periodic units. Harmonic vibrational frequency analysis was conducted to affirm the absence of imaginary frequencies, indicating that the optimized structures represent minima on their respective potential energy surfaces. In addition, this analysis facilitated the assessment of the zero-point energy (ZPE). Quantum mechanics calculations using the Density Functional Theory (DFT) method at the B3LYP/6-31 + G**/LANL2DZ level were performed to unravel the adsorption mechanism, using the Gaussian 16 software package. [^1^](https://www.nature.com/articles/s41467-024-55156-3#ref-CR61) Further characterization of the interaction nature between nitrogen and sulfur as ligands and gold involved natural bond orbital analysis, topological analysis of the electron density using the atoms-in-molecules (AIM) theory, and energy decomposition analysis.[^2^](https://www.nature.com/articles/s41467-024-55156-3#ref-CR62) These analyses collectively reveal that the L–Au bonds exhibit a discernible degree of covalent character. Furthermore, to assess the applicability of fundamental DFT-based electronic structure principles such as the maximum hardness and minimum electrophilicity principles, an examination of the variations in key global reactivity descriptors, namely, chemical hardness (*η*) and chemical potential (*μ*), has been undertaken. [^3^](https://www.nature.com/articles/s41467-024-55156-3#ref-CR63) The evaluation used a model structure comprising a single unit cell of either TTF-COF or TPE-COF. The interaction energy (*E*_int_) was quantified using two distinct methods. In the initial approach, the Eq. ([1](https://www.nature.com/articles/s41467-024-55156-3#Equ5)) was used:

E_int_=E_conf_−(E_COF_+E_Au(III)_) (1)

where *E*_conf_, *E*_COF_, and *E*_Au(III)_ represent the energy of the COF adsorbed with Au(III), isolated COF, and Au(III) after geometry optimization, respectively. This methodology enables a detailed exploration of the alterations in electronic structure and reactivity descriptors, shedding light on the conformity of these systems with established principles in DFT-based theoretical frameworks.

In the second approach, electron density analysis was conducted at the MP2/cc-PVTZ level, using all-electron WTBS basis sets for Au through the Multiwfn software[^4^](https://www.nature.com/articles/s41467-024-55156-3#ref-CR64). This analysis offers valuable insights into the bonding nature of Au.





Figure S1. FT-IR spectra of (a) HATP-COF-1 (blue), [1,1'-biphenyl]-4,4'-diamine (red), and 4,4',4'',4''',4'''',4'''''-(diquinoxalino[2,3-a:2',3'-c]phenazine-2,3,8,9,14,15-hexayl)hexabenzaldehyde (black);(b) HATP-COF-2 (blue), 5,5'-diamino-2,2'-bipyridine (red) and 4,4',4'',4''',4'''',4'''''-(diquinoxalino[2,3-a:2',3'-c]phenazine-2,3,8,9,14,15-hexayl)hexabenzaldehyde (black).





Figure S2. ^13^C Solid NMR spectra of (a) HATP-COF-1 and (b) HATP-COF-2.





Figure S3. TGA curves of (a) HATP-COF-1 and (b) HATP-COF-2.





Figure S4. FE SEM images of (a) HATP-COF-1 and (b) HATP-COF-2.





Figure S5. EDS mapping images of (a) HATP-COF-1 and (b) HATP-COF-2.





Figure S6. Full XPS spectrum of (a) HATP-COF-1 and (b) HATP-COF-2.





Figure S7. (a) PXRD patterns and (b) unit cell in AB-model of HATP-COF-1.





Figure S8. (a) PXRD patterns and (b) unit cell in AB-model of HATP-COF-2.





Figure S9. (a) Nitrogen sorption isotherms and (b) pore size curve of HATP-COF-1.





Figure S10. (a) Nitrogen sorption isotherms and (b) pore size curve of HATP-COF-2.





Figure S11. PXRD patterns of (a) HATP-COF-1 and (b) HATP-COF-2 (as-synthesis: black; HCl: red; water: blue; DMF: green; NaOH: purple).





Figure S12. FT-IR spectra of (a) HATP-COF-1 and (b) HATP-COF-2 (as-synthesis: black; HCl: red; water: blue; DMF: green; NaOH: purple).





Figure S13. (a) Langmuir and (b) Freundlich adsorption isotherm models of HATP-COF-1.





Figure S14. (a) Langmuir and (b) Freundlich adsorption isotherm models of HATP-COF-2.





Figure S15. (a) Pseudo-first-order and (b) pseudo-second-order kinetic models of HATP-COF-1.





Figure S16. (a) Pseudo-first-order and (b) pseudo-second-order kinetic models of HATP-COF-2.





Figure S17. Removal eﬃciency of Au^3+^ for the HATP-COF-1 under diﬀerent pH conditions.





Figure S18. Removal eﬃciency of Au^3+^ for the HATP-COF-1 under various anionic effect.





Figure S19. Removal eﬃciency of Au^3+^ for the HATP-COF-1 under diﬀerent alkali and alkaline conditions.





Figure S20. Removal eﬃciency of Au^3+^ for the HATP-COF-1 under diﬀerent metal ions conditions.





Figure S21. Capture eﬃciency of metal ions using HATP-COF-1 from CPUs.





Figure S22. Cycle performance of (a) HATP-COF-1 and (b) HATP-COF-2 for the Au^3+^ capture.





Figure S23. PXRD patterns of (a) regenerated HATP-COF-1 and (b) regenerated HATP-COF-2.





Figure S24. FT-IR spectra of (a) as-synthesized/regenerated HATP-COF-1 and (b) as-synthesized/regenerated HATP-COF-2.





Figure S25. SEM image of (a) HATP-COF-1 and (b) HATP-COF-2 after repeated cycling.





Figure S26. XPS spectra of Au@HATP-COF-1: (a) full spectrum, (b) N 1s spectra, and (c) Au 4f spectra.





Figure S27. XPS spectra of Au@HATP-COF-2: (a) full spectrum, (b) N 1s spectra, and (c) Au 4f spectra.





Figure S28. PXRD patterns for (a) Au@HATP-COF-1 and (b) Au@HATP-COF-2.





Figure S29. EDS mapping images of Au@HATP-COF-1.





Figure S30. EDS mapping images of Au@HATP-COF-2.

**Table S1**. Element analysis of HATP-COFs.

| Sample information | | C (%) | H (%) | N (%) |
| --- | --- | --- | --- | --- |
| HATP-COF-1 | theoretical | 84.28 | 4.16 | 11.56 |
|  | observed | 82.07 | 5.35 | 11.23 |
| HATP-COF-2 | theoretical | 79.00 | 3.73 | 17.27 |
|  | observed | 77.18 | 4.74 | 16.66 |

**Table S2**. Atomic coordinates of HATP-COF-1 (*P*3, a = 33.22473 Å, b = 33.22473 Å, c = 4.70489 Å, and α = β = γ = 90°, *R*_wp_ =1.76%, *R*_p_ = 1.26%).

| N | 0.01857 | 0.29493 | 0.27287 |
| --- | --- | --- | --- |
| N | 0.28069 | 0.27891 | 0.43346 |
| C | 0.80499 | 0.11628 | 0.09455 |
| C | 0.84604 | 0.12621 | 0.23681 |
| C | 0.8903 | 0.16986 | 0.18672 |
| C | 0.93213 | 0.16908 | 0.17671 |
| C | 0.84455 | 0.09482 | 0.44025 |
| C | 0.89183 | 0.21289 | 0.18121 |
| C | 0.93457 | 0.25434 | 0.19066 |
| C | 0.97599 | 0.25314 | 0.20925 |
| C | 0.9746 | 0.21033 | 0.18791 |
| C | 0.76328 | 0.07531 | 0.15334 |
| C | 0.76226 | 0.04407 | 0.35791 |
| C | 0.80313 | 0.0543 | 0.49937 |
| H | 0.93524 | 0.28718 | 0.20317 |
| H | 1.00635 | 0.20877 | 0.19145 |
| H | 0.7324 | 0.06864 | 0.0396 |
| H | 0.80271 | 0.0308 | 0.65908 |
| C | 0.28918 | 0.6627 | 0.90633 |
| C | 0.29302 | 0.62262 | 0.90732 |
| N | 0.3254 | 0.74545 | 0.8815 |
| C | 0.28323 | 0.74203 | 0.84862 |
| C | 0.24311 | 0.698 | 0.84274 |
| N | 0.24701 | 0.6595 | 0.87471 |
| C | 0.50164 | 0.72001 | 0.80117 |
| C | 0.5409 | 0.76282 | 0.73225 |
| C | 0.53677 | 0.80356 | 0.72004 |
| C | 0.49369 | 0.79987 | 0.78371 |
| C | 0.57419 | 0.84938 | 0.61656 |
| C | 0.58351 | 0.76175 | 0.65263 |
| C | 0.61644 | 0.87407 | 0.76025 |
| C | 0.65079 | 0.91747 | 0.66586 |
| C | 0.64274 | 0.93853 | 0.43287 |
| C | 0.59911 | 0.91576 | 0.30198 |
| C | 0.56542 | 0.87125 | 0.39 |
| C | 0.58205 | 0.73303 | 0.43059 |
| C | 0.62083 | 0.72859 | 0.36828 |
| C | 0.66206 | 0.75387 | 0.52284 |
| C | 0.66289 | 0.78111 | 0.75022 |
| C | 0.6235 | 0.78362 | 0.81991 |
| C | 0.67978 | -0.01633 | 0.3254 |
| C | 0.70462 | 0.7526 | 0.45524 |
| H | 0.50396 | 0.68862 | 0.80512 |
| H | 0.48977 | 0.83031 | 0.77395 |
| H | 0.6222 | 0.86164 | 0.95627 |
| H | 0.68327 | 0.93497 | 0.78062 |
| H | 0.59162 | 0.93171 | 0.12581 |
| H | 0.53229 | 0.85393 | 0.28239 |
| H | 0.55077 | 0.71386 | 0.3064 |
| H | 0.61889 | 0.70642 | 0.19535 |
| H | 0.6936 | 0.7992 | 0.88091 |
| H | 0.62369 | 0.80038 | 1.01449 |
| H | 0.67242 | 0.00011 | 0.15195 |
| H | 0.73599 | 0.77574 | 0.57086 |
| H | 0.87531 | 0.10211 | 0.55889 |
| H | 0.20449 | 0.06809 | 0.17278 |
| H | 0.86006 | 0.66536 | -0.06351 |
| H | 0.78555 | 0.64564 | 0.18817 |

**Table S3**. Atomic coordinates of HATP-COF-2 (*P*3, a = 34.71496 Å, b = 34.71496 Å, c = 4.71199 Å, and α = β = γ = 90°, *R*_wp_ = 1.80, *R*_p_ = 1.44%).

| N | 0.00914 | 0.28171 | 0.42396 |
| --- | --- | --- | --- |
| N | 0.2594 | 0.27445 | 0.45711 |
| N | 0.81394 | 0.10206 | 0.27373 |
| C | 0.84759 | 0.10947 | 0.45668 |
| C | 0.89049 | 0.15368 | 0.4539 |
| N | 0.92765 | 0.16195 | 0.62034 |
| C | 0.84037 | 0.07491 | 0.64488 |
| C | 0.89432 | 0.18728 | 0.28362 |
| C | 0.93203 | 0.22821 | 0.27186 |
| C | 0.96927 | 0.23801 | 0.44022 |
| C | 0.96651 | 0.2036 | 0.61257 |
| C | 0.77423 | 0.0628 | 0.26487 |
| C | 0.7664 | 0.02756 | 0.44868 |
| C | 0.8002 | 0.03454 | 0.63853 |
| H | 0.93296 | 0.25335 | 0.13178 |
| H | 0.99442 | 0.20805 | 0.73874 |
| H | 0.74922 | 0.0594 | 0.11469 |
| H | 0.79522 | 0.00844 | 0.78539 |
| C | 0.29226 | 0.66635 | 0.51342 |
| C | 0.29258 | 0.62557 | 0.51486 |
| N | 0.33162 | 0.7462 | 0.51793 |
| C | 0.29233 | 0.74654 | 0.51546 |
| C | 0.25217 | 0.70603 | 0.50664 |
| N | 0.25316 | 0.66708 | 0.50752 |
| C | 0.49513 | 0.70839 | 0.52242 |
| C | 0.53655 | 0.74883 | 0.51526 |
| C | 0.53633 | 0.79031 | 0.49062 |
| C | 0.49439 | 0.78838 | 0.49295 |
| C | 0.57722 | 0.83579 | 0.43733 |
| C | 0.57814 | 0.74538 | 0.53713 |
| C | 0.61479 | 0.85473 | 0.6159 |
| C | 0.65158 | 0.89699 | 0.56598 |
| C | 0.65133 | 0.92386 | 0.34501 |
| C | 0.61289 | 0.90687 | 0.17561 |
| C | 0.5767 | 0.86347 | 0.21909 |
| C | 0.58669 | 0.71852 | 0.35189 |
| C | 0.62643 | 0.71667 | 0.37324 |
| C | 0.65824 | 0.74138 | 0.58275 |
| C | 0.64751 | 0.7647 | 0.78095 |
| C | 0.60702 | 0.76424 | 0.76701 |
| C | 0.69025 | -0.03045 | 0.29627 |
| C | 0.70203 | 0.74313 | 0.61251 |
| H | 0.49472 | 0.67703 | 0.53671 |
| H | 0.49317 | 0.81891 | 0.4805 |
| H | 0.61531 | 0.83957 | 0.81037 |
| H | 0.67953 | 0.90939 | 0.71052 |
| H | 0.61102 | 0.92692 | 0.00604 |
| H | 0.54818 | 0.85143 | 0.0797 |
| H | 0.56306 | 0.69975 | 0.18625 |
| H | 0.63258 | 0.69673 | 0.22325 |
| H | 0.66997 | 0.78225 | 0.95451 |
| H | 0.59724 | 0.777 | 0.94459 |
| H | 0.68874 | -0.01054 | 0.12468 |
| H | 0.7209 | 0.759 | 0.80199 |
| H | 0.21251 | 0.13269 | 0.80289 |
| H | 0.31656 | 0.13468 | 0.143 |

**Reference**

1. Frisch, M. J. et al. Gaussian 16 Revision C.01 (Gaussian Inc., 2016).
2. Matta, C. F. & Boyd, R. J. The Quantum Theory of Atoms in Molecules: From Solid State to DNA and Drug Design (Wiley-VCH Verlag GmbH & Co. KGaA, 2007).
3. Pearson, R. G. Absolute electronegativity and hardness correlated with molecular orbital theory. *Proc. Natl. Acad. Sci.* 1986, 83, 8440-8441.
4. Lu, T. & Chen, F. Multiwfn: A multifunctional wavefunction analyzer. *J. Comput. Chem*. 2012, 33, 580-592.
